# Community capability building for environmental conservation in Lake Biwa (Japan) through an adaptive and abductive approach

**DOI:** 10.1007/s42532-021-00078-3

**Published:** 2021-03-29

**Authors:** Yasuhisa Kondo, Eiichi Fujisawa, Kanako Ishikawa, Satoe Nakahara, Kyohei Matsushita, Satoshi Asano, Kaoru Kamatani, Satoko Suetsugu, Kei Kano, Terukazu Kumazawa, Kenichi Sato, Noboru Okuda

**Affiliations:** 1 Research Institute for Humanity and Nature, Kyoto, Japan; 2 Biwako-Chishin, Otsu, Shiga Japan; 3 Ohmi Data Institute, Otsu, Shiga Japan; 4 Lake Biwa Environmental Research Institute, Otsu, Shiga Japan; 5 Faculty of Economics, Shiga University, Hikone, Shiga Japan; 6 Graduate School of Global Environmental Studies, Kyoto University, Kyoto, Japan; 7 College of Gastronomy Management, Ritsumeikan University, Kusatsu, Shiga Japan; 8 Faculty of Education, Shiga University, Otsu, Shiga Japan; 9 Faculty of Life Sciences, Kyoto Sangyo University, Kyoto, Japan; 10 Research Center for Inland Seas, Kobe University, Kobe, Hyogo Japan

**Keywords:** Transdisciplinary action research, Adaptive abduction circuit, Area capability, Ethical issues, Ecological conservation, Citizen-driven environmental governance

## Abstract

In the south basin of Lake Biwa, Shiga, Japan, overgrown aquatic weeds (submerged macrophytes) impede cruising boats and cause unpleasant odors and undesirable waste when washed ashore. To address this socio-ecological problem, Shiga Prefectural Government implemented a public program to remove overgrown weeds and compost them ashore to conserve the lake environment, while coastal inhabitants and occasional volunteers remove weeds from the beaches to maintain the quality of the living environment. However, these effects are limited because of disjointed social networks. We applied an adaptive and abductive approach to develop community capability to jointly address this problem by sharing academic knowledge with local actors and empowering them. The initial multifaceted reviews, including interviews and postal questionnaire surveys, revealed that the agro-economic value of composted weeds declined in historical and socio-psychological contexts and that most of the unengaged public relied on local governments to address environmental problems. These findings were synthesized and assessed with workshop participants, including local inhabitants, governmental agents, businesspeople, social entrepreneurs, and research experts, to unearth the best solution. The workshops resulted in the development of an e-point system, called *Biwa Point*, to promote and acknowledge voluntary environmental conservation activities, including beach cleaning. It may contribute to enhancing the socio-ecological capability of communities. Additionally, ethical issues, such as publication of inconvenient truths, undesired interpretation by the researchers, and social constraints in research methods, arose through our research practice.

**Supplementary Information:**

The online version contains supplementary material available at 10.1007/s42532-021-00078-3.

## Introduction to a recent socio-ecological problem in Japan

Environmental deterioration can result from damaging interactions between human societies and ecosystems. This is often perceived as a “wicked problem” that has no clear-cut solution (Norris et al. 2016, p.115; Rittel and Webber 1973, p.160). Such a problem cannot simply be solved by research experts; rather, it requires team-based collaboration with experts from different domains for interdisciplinary research (Kelly et al. 2019, p.150; Repko and Szostak 2020, pp.6–9), and with practitioners based at governments, funding bodies, industry, non-profit organizations (NPOs), and civil society for transdisciplinary research (OECD 2020, p.4; Pohl et al. 2017, pp.319–323). Interdisciplinary and transdisciplinary research can help “dissolve” the problems, which may be perceived differently by different actors. Due to the nature of the problems and the dynamics of diverse collaborators, the process of team-based research on socio-ecological problems is not always as linear as conventional scientific research, in particular because it may experience some tortuous adaptations in its methodological development. This study presents our experience of an adaptive and abductive approach to address a socio-ecological problem in Lake Biwa, Japan.

### The problem: overgrown aquatic weeds in Lake Biwa

**Fig. 1:**
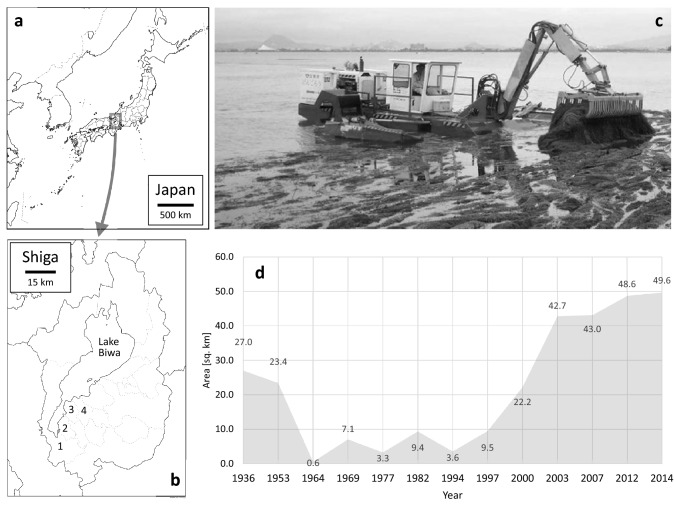
General information on the overgrown aquatic weeds in Lake Biwa. **a** Location of Lake Biwa in Japanese Archipelago (base map: https://www.freemap.jp/); **b** Location of Lake Biwa and municipalities in the south basin area, Shiga prefecture. 1: Ōtsu; 2: Kusatsu; 3: Moriyama; 4: Yasu (base map: https://www.freemap.jp/); **c** A specially manufactured workboat removes overgrown aquatic weeds in the south basin of Lake Biwa (courtesy of the Lake Biwa Environmental Conservation Division of Shiga Prefectural Government); **d** Diachronic fluctuation of submerged macrophytes in the south basin of Lake Biwa (after Haga 2015, Table 1; 2020, Table 1). The vertical axis represents the area of the lake surface occupied by submerged macrophytes, and the horizontal axis represents the year of survey

Lake Biwa (*Biwako* locally) is the largest freshwater lake (area: 670 km^2^) in Japan, located in Shiga Prefecture in the central part of Honshū Island (Fig. 1a, b; Kawanabe et al. 2020, p.ix). From ecological and geographical viewpoints, the lake consists of a mesotrophic north basin (*Hokko*; mean depth: 43 m; area: 618 km^2^) and a eutrophic south basin (*Nanko*; mean depth: 3.5 m; area: 52 km^2^). The littoral zone of the south basin is an urbanized area (servicing cities of Ōtsu, Kusatsu, Moriyama, and Yasu, from west to east; see Fig. 1b) with a population of approximately 630,000 people, connected to the urban belt including Kyoto, Osaka, and Kobe. Lake Biwa currently supplies potable water to 17 million people in this belt.

The water quality of Lake Biwa, particularly in the south basin, has significantly worsened since the late 1960s due to population growth and industrial developments (International Lake Environment Committee Foundation 2014, p.100). Eutrophication caused the first large-scale outbreak of red tide (i.e., overgrowth of plankton) in 1977 (*Ibid.*, p.100). To reduce the discharge of phosphorus contained in synthetic detergents, local homemakers launched the “Soap Movement,” which led to the enforcement of the Ordinance for the Prevention of Eutrophication of Lake Biwa in 1980 (*Ibid*., p.92). Under this ordinance, the public sewerage systems were improved. Since then, the water quality has gradually recovered, but cyanobacterial water blooms have been observed since 1983 (*Ibid*., pp.100, 110).

In the south basin, aquatic weeds (or submerged macrophytes in biological and ecological terms), including endemic species like *Potamogeton maackianus* (*sen’nin-mo* locally) and *Hydrilla verticillata* (*kuromo*) and exotic species like *Elodea nuttallii* (*kokanadamo*) and *Egeria densa* (*ōkanadamo*), have been proliferating annually since a substantial decrease in lake water in 1994 (Haga 2020, pp.294–296; Hamabata et al. 2020, pp.105–113). This coincided with a remarkable increase in water transparency, which suggested a so-called ecological regime shift (Hamabata et al. 2020, pp.105–113; Ishikawa and Okamoto 2015, pp.488–493). In 2014, weeds occupied an area comprising 95% (49.6 km^2^) of the south basin (Fig. 1d; Haga 2020, Table 1).

**Table 1:** Four multi-actor workshops for the synthesis and assessments to find a better solution to the overgrown aquatic weed problem in Lake Biwa

No	Month/Year	Venue	Number and attribute of participants	Major outcomes
1	11/2017	Kusatsu	12 (local NPO members, prefectural government and city officers)	To use composted weeds for planters at school, and gift them from graduates to new pupils
2	4/2018	Ōtsu	14 (Local NPO members, social entrepreneurs)	To make a herbarium using endogenous weeds at school
3	7/2018	Ōtsu	28 (social entrepreneurs, city officers, businesspeople)	To develop a point system to acknowledge voluntary beach cleaning and compost making
4	8/2018	Ōtsu	8 (student, fisherfolk, prefectural officers, flower shop owner, social entrepreneurs)	To change people's mind from “joyful “ (to join beach cleaning events) to “want” (Biwa Points as token of goodwill)

Overgrown weeds impede cruising boats. The Shiga Prefectural Government, responsible for the ecosystem management of Lake Biwa, spent 330 million yen (~ 3.1 million US dollars) on a public conservation program in 2016 (Shiga Prefectural Government 2017) to mitigate the harmful effects of overgrown weeds. The gross annual budget, including funds for removing designated alien species, was approximately 600 million yen (*Ibid.*, pers. comm.). In collaboration with the affiliated Ohmi Environment Conservation Foundation, the prefectural government mows aquatic weeds offshore using special boats and devices (Fig. 1c), but the removed mass (approximately 5200 tons) was less than 30% of the estimated total standing stock (18,173 tons; Haga 2015, Table 1) in 2014.

The mown weeds are transported to a dumpsite, where they are dried and fermented for two summers. A soil microbial study demonstrated the positive effects of composted weeds on terrestrial plant growth (Matsuoka et al. 2020, p.451)*.* The composted weeds are provided, free of charge, to locals. The prefectural office also financially supports academic and private sectors to develop technologies to prevent aquatic weed overgrowth.

Aquatic weeds cause unpleasant odors and undesirable waste when washed ashore, particularly in the summer. Occasionally, inhabitants request local municipal offices to remove decayed weeds. Under the Waste Management Law, municipal offices are responsible for clearing weeds from public areas. In 2016, the Ōtsu City Office received 15 complaints and spent approximately 23 million yen (~ 0.22 million US dollars) on cleaning and disposal of weed waste (Suihōzan 2017, p.4). Cleaning activities rely on coastal residents and occasional volunteers, and therefore, their effect is limited.

### Background characteristics of “communities” in Japan

In Japan, local communities (such as the present-day *jichikai* and *chōnaikai* residential associations) are traditionally delimited by living places, and each inhabitant of the area must become a member. Such communities administer communal events such as rituals, festivals, fire prevention, public space cleaning, and estate and heritage management (Braibanti 1948, pp.140–154). The present form of these communities stems from the premodern Edo period (1603–1868 AD).

The recent rise of individualism along with an aging and decreasing population has weakened the solidarity and functionality of local communities. In such a shrinking community, the information divide between conventional association members (typically elderly inhabitants) and newer inhabitants (typically younger individuals) may cause a serious social divide and produce “shadow communities” that are hardly recognized from the exterior.

## Action research with an adaptive and abductive approach

We recognized this complex of socio-ecological problems in 2016. With the financial aid from a private foundation, a transdisciplinary research team including academic scholars such as ecologists, historians, anthropologists, sociologists, economists, and science communication experts, as well as practitioners such as social entrepreneurs and workshop facilitators, was launched in 2017.

### Methodological foundation

This team took action research as its methodological foundation. Originally defined by Kurt Lewin as “research which will help the practitioner” (Lewin 1946, p.34; see also Adelmann 1993, pp.7–8), action research is recognized as “a participatory, democratic process concerned with developing practical knowing in the pursuit of worthwhile human purposes, grounded in a participatory worldview” (Brydon-Miller et al. 2003, pp.10–11; Reason and Bradbury 2001, p.1). This means that collaborative knowledge production through transdisciplinary action research is not always linear as conventional scientific research. Rather, an abductive approach may be applied to find an ad hoc best-fit hypothesis as a pathway to a solution (Stigendal and Novy 2018, p.213; Yonemori 2007, pp.53–72).

**Fig. 2:**
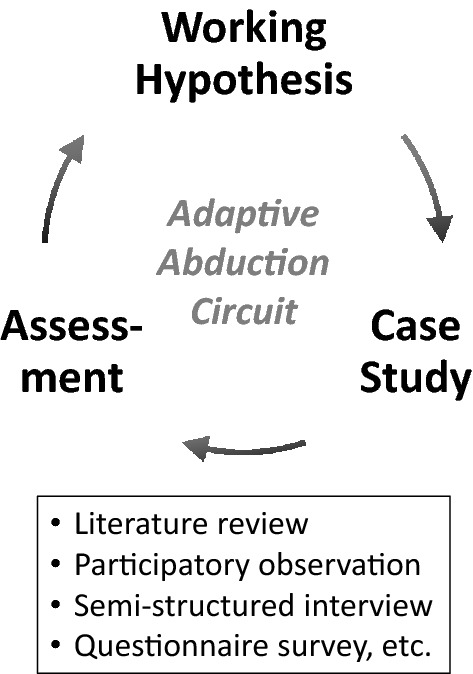
Adaptive abduction circuit as an iterative process of working hypothesis, case study, and assessment conducted using conventional social survey methods such as participatory observation, semi-structured interview, and questionnaire survey

Our action research was improved through an adaptive abduction circuit, comprising an iterative process of working hypothesis, case study, and assessments (Fig. 2). The working hypothesis must be improved by assessing practical case studies in a shorter cyclic period than in the prevalent Plan-Do-Check-Act (PDCA) cycle (Fouché and Brent 2020, p. 8). Conventional social survey methods, such as literature review, participatory observation, semi-structured interview, and questionnaire survey, are used in assessments.

**Fig. 3:**
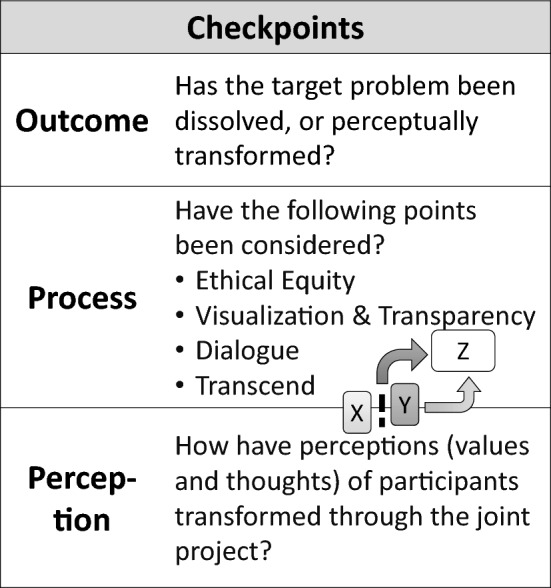
Checkpoints for research outcome, process, and perceptual transformation of participants (adapted from Kondo et al. 2019: Fig. 4). The diagram in the middle of the checkpoint table shows a process of transcend, in which actors with different values and thoughts (*X* and *Y*) find path(s) to build a shared platform to work together toward target issues (*Z*)

The checkpoints for assessment were classified into research outcome, process, and perceptual transformation of participants (Fig. 3; European Commission 2015, pp. 22–23). The outcome of an action research project can be assessed by checking if the target problem has been dissolved or perceptually transformed. The process is checked for ethical equity among actors, visualization and transparency, dialogue, and transcend are properly considered (Kondo et al. 2019, pp. 57–58). In transcend, actors with different values and thoughts (*X* and *Y* in Fig. 3) find path(s) to build a shared platform to work together toward target issues (*Z*). It is also important to assess how the perceptions, values, and thoughts of participants transform during the project. These checkpoints are particularly recommended for self-assessment in case participants face obstacles during action research.

**Fig. 4:**
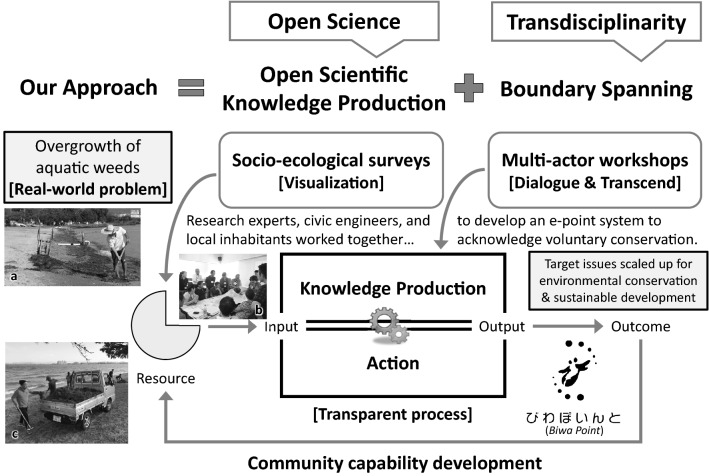
Open team science approach and community capability cycle to develop a local community and acknowledgement system (Biwa Point) to address the aquatic weed issues in Lake Biwa (adapted from Kondo et al. 2019: Fig. 2). Photograph **a** A local volunteer removes drifted waterweed from the shore in front of his guesthouse (courtesy of Eiji Yamada); **b** A workshop in Ōtsu in July 2018, with 28 participants including businesspersons, civic engineers, governmental employees, inhabitants, and research experts; **c** A group beach cleaning held in Ōtsu in November 2019. The *Biwa Point* system was tested during this event

Inspired by the open science policy of making primary outputs of publicly funded research publicly accessible (OECD 2015, p. 7), we tried to make our action research process as open as possible. Hence, practitioners and local actors were empowered to autonomically address problems. The concept of open science was extended to open scientific knowledge production systems, interlinked with the concept of boundary spanning, or connecting to other actors across socio-psychological gaps, as an essence of transdisciplinary approach (Fig. 4; Kondo et al. 2019, pp. 57–59).

### Practical application workflow

We conducted two rounds of the abduction circuit. The first round began with the working hypothesis that it was necessary to combine individual efforts to address the overgrown weed problem. As a case study, the current issues were reviewed from multifaceted academic viewpoints, including an ecological interpretation (see Sect. 3.1), interviews and actor analysis (Sect. 3.2), a questionnaire survey (Sect. 3.3), and a historical study (Sect. 3.4). The methods for individual work packages are described in the relevant subsections.

The preliminary results of these multifaceted reviews were synthesized and assessed in multi-actor workshops to improve the working hypothesis in developing a better solution to the problems in the second round (Fig. 4; see also Sect. 4). These workshops functioned as a transparent process for knowledge co-production, action, and social networking to develop a community to share and address the problem together. The methods and results of the workshops are presented in Sect. 4, and the implications are discussed from the viewpoints of community capability, social innovation, and ethical matters in Sect. 5.

## Multifaceted surveys to understand the current problem

### An ecologist’s view: ecological regime shift and needs for adaptive management

According to literature in theoretical ecology, alternative stable states exist in lake ecosystems because ecosystem responses to environmental factors are nonlinear (Carpenter 2003, p.179; Lewontin 1969, pp.13–24). In shallow eutrophic lakes, there are “turbid states” and “clear states” in which phytoplankton and submerged macrophytes dominate, respectively. When the effect of an environmental change is greater than a certain threshold, a regime shift to another state occurs. Moreover, in the turbid state, when phytoplankton dominates, light cannot penetrate to the bottom of the lake, which causes aquatic plants to die and increases the populations of invertebrates, Cyprinidae fish planktivores, and smaller sized zooplankton that maintains the lake in a turbid state.

In the clear state, aquatic plants dominate, and plant roots restrain the resuspension of mud during wind events. Submerged macrophytes compete with phytoplankton for nutrients and release some allelochemicals, thereby inhibiting the growth of phytoplankton. Many attached aquatic microorganisms obtain food and shelter from the macrophytes and some decompose senescent macrophytes, which increases lake transparency (Jeppesen et al. 1998, p.423). Hence, it is theoretically difficult to achieve a utopian-like condition—the clear water that many residents prefer does not sustain large fish populations.

To complicate things further, global warming and climate change have caused unprecedented events that affect clear water states, such as increased temperatures and rainfall, violent typhoons, and phytoplankton blooming in the north basin, which now practically occurs annually. Due to drastic environmental fluctuations, the lake can easily return to a “turbid state.”

In recent years, “adaptive management” has increasingly been applied to the management of ecosystems under uncertain conditions (Allen et al. 2012, pp. 3342–3357). In adaptive management, the strategy may be changed by the feedback from monitored results. In the case of overgrown macrophytes in Lake Biwa, since the prefectural program has not yielded enough profit to warrant a business, it will not be practical to continuously provide for a mowing budget in the future (see also Sect. 3.3). However, the cost of burning mown weeds and handling wastes is slightly decreased by composting. Therefore, an adaptive management system to discover a threshold value for regime shift is needed (Ishikawa et al. 2019, pp. 69–82; Ishikawa et al. 2020, pp. 577–581).

### Interviews and actor analysis revealed a multi-actor situation

We conducted interviews with approximately 30 local actors identified by practitioners. Interviewees included officers of the Lake Biwa Environmental Conservation Division of Shiga Prefectural Government and environmental departments of Ōtsu and Moriyama cities, local inhabitants, farmers, fisherfolk, student volunteers, businesspeople, and social entrepreneurs. Interviews mainly focused on the overgrown weed problem, but related topics and thoughts on their living and/or working situations were also discussed extemporaneously. Also, 80 local inhabitants were interviewed about the usage of composted weeds on the occasion of free distribution in February 2018. The results of interviews were crosschecked with participatory observations at meetings, workshops, and field events associated with the overgrown weed problem. Based on these interviews and observations, local actors were qualitatively classified as discussed below.

#### Different perceptions among actors

**Fig. 5:**
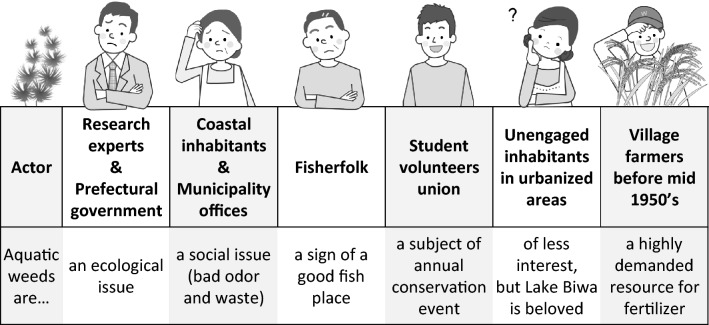
Major actors and their values and thoughts on the aquatic weeds of Lake Biwa

The interviews revealed that the perception and understanding of the weed problem differed among actors, although they shared the recognition that it was a wicked socio-ecological problem (Fig. 5). The prefectural government is responsible for lake conservation and addressed the problem of overgrown aquatic weeds in terms of ecosystem management, which was also emphasized by ecologists as discussed in the previous subsection. In contrast, coastal inhabitants and municipality officers tended to regard this issue as a social problem of unpleasant odor and waste, which were perceived as a nuisance.

In addition to local governments and coastal inhabitants, local fisherfolk and student volunteers were identified as stakeholders. For fisherfolk, aquatic weeds were a sign of a good fishing place, just as they were in the Edo period (see Sect. 3.4). However, catch of endemic species, such as *Carassius buergeri grandoculis* (*nigoro-buna* locally) and *Gnathopogon caerulescens* (*hon-moroko*), drastically decreased. In recent years, fisherfolk have contributed to the prefecture’s program by removing aquatic weeds (Fig. 1c).

Meanwhile, a non-profit student organization, called the International Students Volunteer Association, coordinated an annual conservation event to remove designated alien weeds such as *Ludwigia grandiflora subsp grandiflora* (*ōbana-mizukinbai*), with some hundred participants. Students participated for fun and networking. They shared information on the ecological issues of alien weeds in Lake Biwa in a pre-event briefing, and local governments provided logistical support, such as waste pick-up.

In addition, a local company funded by the prefectural government commercialized composted weeds as a product. The funding program also supported a local craft shop to use dried and milled weeds for the coloring of glasswork. A flower shop in Ōtsu sold herb kits with composted weeds. Furthermore, a confectionery started using composted weeds to grow plants for shop display.

#### Personal activities and launch of a civic group

Interviews also identified some local individuals who had been personally clearing washed weeds from the shore. For instance, an individual (Y-san) has been cleaning Manohama beach in front of his guesthouse in Katata, Ōtsu, every morning alone (Fig. 4a) since 2014, and sharing dried weeds with those who desired them since 2016. Neighboring residents, including M-san, received weeds for their home gardens to grow vegetables for family consumption. These activities were undertaken for their own purposes (*ikigai*) and did not count as ecological conservation. Y-san stated, “I did nothing. I just cleaned up the beach in front of my home.” M-san said, “I do not know the aquatic weed problem well as we are just growing vegetables.”

Y-san posted online about beach cleaning and almost daily. W-san, an environmental sociologist in residence, recognized Y-san through these daily posts. W-san also knew of a local corporate executive, K-san, who proposed an idea to acknowledge and circulate “a goodwill” to Lake Biwa (a prototype of the *Biwa Point* presented later). W-san invited Y-san and K-san to establish a civic group called *Suihōzan* (meaning aquatic weeds are a gold mine), launched in late 2017 (Wakita et al. 2020, pp. 203–212).

One of the authors (Fujisawa) also joined this group. He has participated in civic projects in collaboration with Ōtsu City before. Fujisawa suggested submitting a proposal on “the civic actions to develop a social system to reuse aquatic weeds in Lake Biwa as resources” with Ōtsu City to a social innovation contest, Challenge Open Governance 2017. This proposal was nominated as a finalist for the award. Through this proposal, *Suihōzan* became a fundamental organization to address the overgrown weed problem, strengthened by the potential of civic collaboration with local governments in the cities of the south basin area.

### Questionnaire survey highlighted less engaged actors

In addition to the aforementioned “active” actors, there was a “small-voice” majority living in urbanized areas. In January 2018, a postal questionnaire survey was conducted in the three municipalities in the south basin area, namely Ōtsu (southern part only), Kusatsu, and Moriyama, to investigate (1) to what degree inhabitants were aware of the presence of and heaviness of aquatic weeds in Lake Biwa, and (2) how they evaluated the measures to tackle this socio-ecological problem. The survey sheets were mailed to 30,203 households in 78 randomly sampled postal code areas. Of those, 4,578 households responded (response rate: 15.1%). The age group of respondents was biased towards elder generations (57.6% were 60 years old or older) in comparison with the actual demography of the study area, because household heads were asked to respond. The gender balance (49.8%, male; 49.5%, female) was close to that of the actual demography.

#### Overall trend

**Fig. 6:**
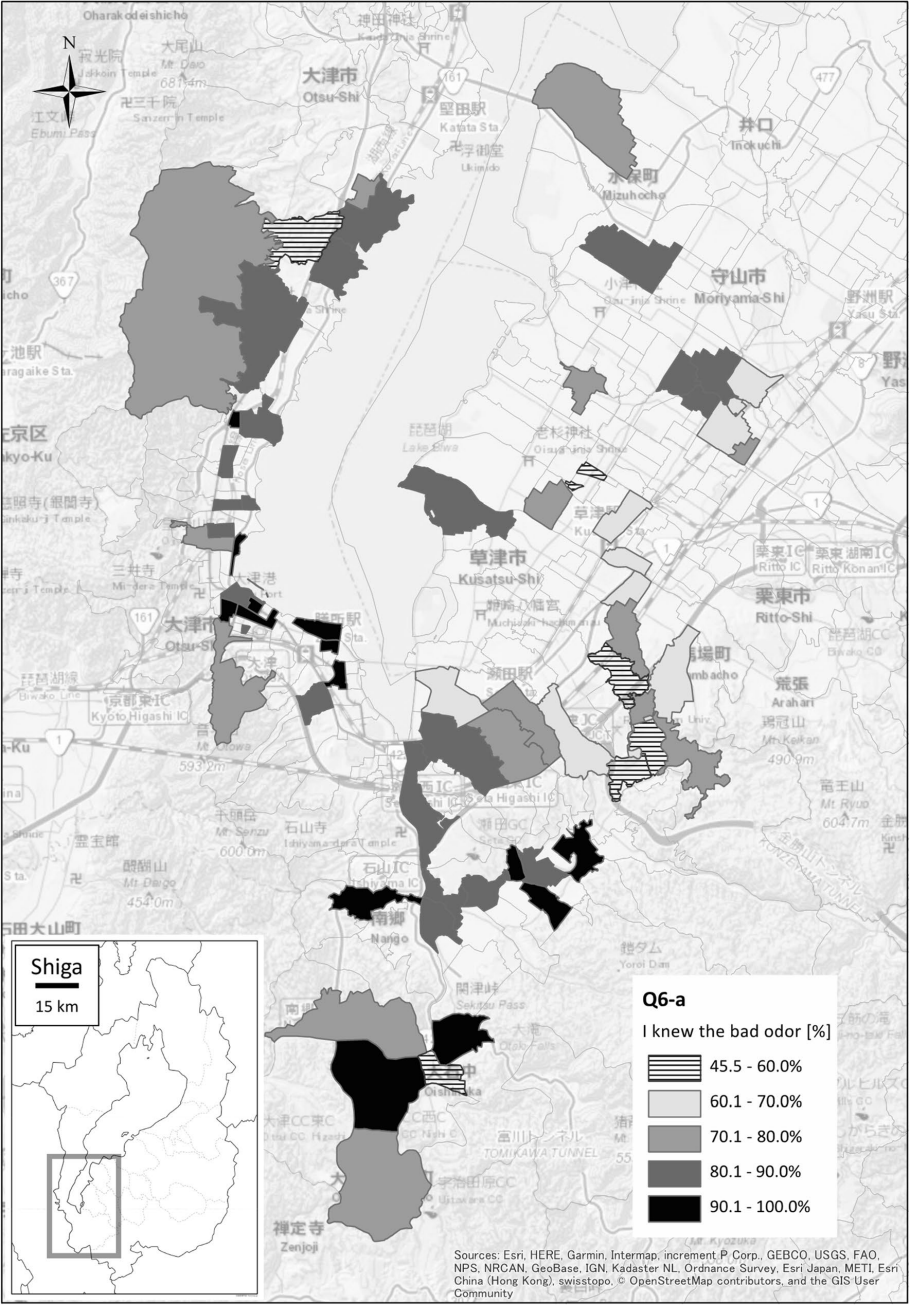
Geographical distribution of the percentage of respondents who knew about the bad odor of drifted weeds in the South Basin of Lake Biwa. Note that 78 areas were randomly sampled out of 622 postal code areas in the study area

Most respondents recognized that overgrown aquatic weeds (1) created an unpleasant odor, (2) blemished lake views, (3) degraded the water quality, and (4) caused undesirable effects on aquatic organisms and fisheries. Since local print and broadcast media had run stories on the weed problem, even respondents who did not live near Lake Biwa and seldom visited the lakeshore were cognizant of the ongoing issues (Fig. 6). In addition, 91.4% of the respondents thought that they benefited from the ecosystem of Lake Biwa, while 67.2% thought that public authorities should measure alien fish populations (asked as a proxy of environmental problem measurements).

**Fig. 7:**
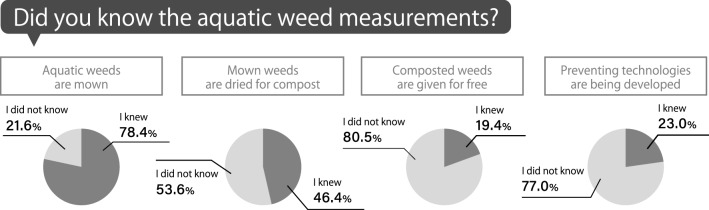
Percentage of respondents who recognized the measurement program coordinated by the Shiga Prefectural Government for the thick growth of aquatic weeds in Lake Biwa per measure (i.e., weed removal, production of composted weeds, occasions to give away composted weeds, and developing technology to prevent the overgrowth of weeds)

Regarding the measurement program coordinated by Shiga Prefectural Government (see Sect. 1), 78.4% of respondents recognized that the prefectural government removed aquatic weeds, while fewer respondents were knowledgeable of the production of composted weeds (46.4%), free distribution of composted weeds (19.5%), and developing technologies to prevent the overgrowth of weeds (23.0%; see Fig. 7).

Further, inhabitants’ priorities regarding governmental actions concerning the aquatic weed problems were different from their recognition rates. Their top priority was mowing, and their second priority was technological development to prevent overgrowth, followed by the free distribution of composted weeds. This indicates that overgrowth prevention was more important for inhabitants than effective reuse.

#### Willingness to pay for aquatic weed measures

In addition to public awareness, the cost–benefit balance of environmental conservation cannot be ignored. Since the prefectural government spends 600 million yen per year on the conservation program, the cost–benefit balance of the budget was evaluated from the viewpoint of the inhabitants. The social benefit of measures tackling overgrown weeds was analyzed using the contingent valuation method, which is universally applied in environmental economics to derive the willingness to pay (WTP) for environmental changes (Phaneuf and Requate 2016, pp. 576–579).

**Fig. 8:**
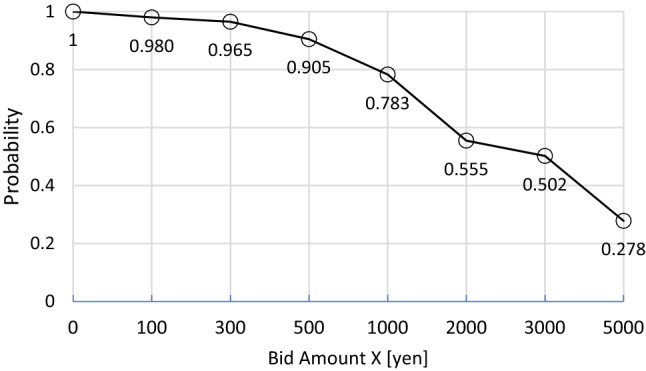
Linear interpolation for calculated probability “yes” against the bid amount Xs to the question “would you be willing to pay *X* yen per year for the environmental conservation to mow overgrown weeds, compost them for free distribution, and support the development of new technologies to prevent the overgrowth of weeds in Lake Biwa?” The vertical axis represents probability “yes” and the horizontal axis represents the presented bid amount *X*, randomly set to 100, 300, 500, 1000, 2000, 3000, or 5000 yen

The dichotomous choice format was applied to elicit respondents’ WTP as follows. First, respondents were asked to answer the hypothesized question “would you be willing to pay X yen per year for the environmental conservation to mow overgrown weeds, compost them for free distribution, and support the development of new technologies to prevent the overgrowth of weeds in Lake Biwa?” where X was the presented bid amount, randomly set to 100, 300, 500, 1000, 2000, 3000, or 5000 yen. Second, the probability of “yes” was calculated against the hypothesized question by the bid amount Xs, excluding resisted answers. Then, a WTP was obtained by multiplying the bid amounts by the corresponding probabilities of “yes” (Fig. 8). Finally, mean WTP was obtained by integrating the probability “yes” function from *X* = 0 to *X*_max_ where probability “yes” equals zero.

It was anticipated that the probability “yes” would get to zero at *X* = 5000 at the stage of setting bid amounts. However, the probability “yes” was much higher than zero, equaling 0.278, at *X* = 5000. Therefore, the mean WTP was underestimated. Taking this limitation into account, the mean WTP was calculated by truncating the probability “yes” distribution at 5000 yen, totaling 2879 yen for the mean WTP per household. This value was slightly higher than the average household expenditure for a weekend leisure activity in the Lake Biwa area (2806 yen). Since the study area had approximately 240,000 households in total, 690 million yen would be collected annually for the measures directed against overgrown weeds. This estimate was higher than the total cost of the actual conservation program (see Sect. 1). It should be noted that income effects and lack of knowledge of preferences might affect the estimates (but see Sect. 5.3).

This cost–benefit analysis supported the environmental conservation program of the prefectural government. However, the value placed on the overgrown weeds varied among local inhabitants, as well as the economic and psychological damages. Different values may result from different priorities within the conservation program. Therefore, if the government were to plan an environmental tax, it would be difficult to even the tax burden based on the beneficiary liability principle. A more effective way to involve local inhabitants and other marginalized actors in decision-making is required.

### Aquatic weeds in Lake Biwa three hundred years ago: a historical foundation

In addition to these “present” actors, “past” actors in the historical context were also identified. Historically, aquatic weeds have long been indispensable natural resources for the human subsistence economy, being used as organic fertilizer, and exploited for popular fishing places, due to their propensity to serve as spawning and nursery grounds for fish. Historical documents evidenced that aquatic weeds were already in high demand by the early eighteenth century (middle Edo period), although the agro-economic value of aquatic weeds sharply declined in the mid-1950s when chemical fertilizers were introduced (Hiratsuka et al. 2006, p. 94).

#### Disputes over weed exploitation in the 1700s

According to an old document possessed by Kanda Jinja (shrine), Ōtsu, a dispute concerning rights over mowing aquatic weeds (called *mogusa* at that time) took place in present-day Ōtsu in 1701. Ichiemon, an inhabitant of Ōtsu Kitaho-chō, filed a suit against the fisherfolk of Honkatata-mura, a coastal village that was part of present-day Katata, Ōtsu. The fisherfolk of Honkatata-mura had collected aquatic weeds in the fishing area over which Ichiemon’s family had enjoyed exclusive rights for several generations.

According to Ichiemon, a previous intrusion had concluded with a verbal negotiation between him and the fisherfolk. However, he instituted a new suit against the fisherfolk because they had again intruded into his fishing area. Refuting Ichiemon’s arguments, the fisherfolk stated two reasons why they should be allowed to use the fishing area: (1) “we have fishing rights over the entirety of Lake Biwa,” and (2) “there was a dispute twenty-five years ago as well, and our rights were approved then.”

This conflict ran as follows: The fisherfolk from Honkatata-mura had been collecting aquatic weeds for farmers to use as fertilizer. For this reason, farmers from Honkatata-mura and the neighboring Imakatata-mura joined the dispute in support of the fisherfolk. Consequently, Honkatata-mura and Imakatata-mura won the suit and acquired the rights to continue collecting aquatic weeds.

#### Local traditional knowledge about aquatic weeds

Local people during the Edo period possessed indigenous knowledge about the agricultural efficacy of aquatic weeds. In one of the documents exchanged during the dispute over weed exploitation, one excerpt described aquatic weeds as “nourishment for rice paddies and fields,” constituting an agricultural fertilizer. This fact indicates that aquatic weeds were already being used as fertilizer at the beginning of the 1700s.

The same document also mentioned that aquatic weeds were used “because there were no grassy hills or meadows in the village.” This suggests that local people regarded aquatic weeds as a lower-quality fertilizer than grass obtained from grassy hills and meadows. Nevertheless, the fact that two villages cooperated to win the rights over aquatic weeds indicates that weeds were a valuable resource for fertilizer, especially for lakeside villages that could produce no other fertilizers of their own.

Ōtsu was already an urbanized area, called “Ōtsu-hyakuchō” (*hyakuchō* roughly means “a hundred town sections”). As such, the wastewater from Ōtsu created a eutrophic condition for aquatic weeds that was favorable for the fisherfolk of Honkatata-mura. It appears that local people at that time had certain knowledge about the conditions of aquatic weed growth and management, as one of the documents recorded fisherfolk from Honkatata-mura explaining that “wastewater from Ōtsu grows aquatic weeds well.”

## Multi-actor workshops for solution-oriented assessments

In collaboration with *Suihōzan*, four local multi-actor workshops were held in 2017 and 2018 (Table 1). Participants included local NPO members, inhabitants, agents of the Shiga Prefectural Government and municipalities, businesspeople, social entrepreneurs, and research experts. The preliminary results of the reviews were shared with participants to visualize the problem (Fig. 4).

A synthetic assessment of survey results illuminated that the overgrown aquatic weeds in the south basin of Lake Biwa were perceived differently in various socio-geographical contexts. There were differences in the perception of conventional stakeholders and the less engaged and marginalized public. This socio-psychological gap was rooted in the historical context and made it difficult to recruit volunteer groups and workers to clean the beach. The vulnerability of social networks made this issue a “wicked problem.” The overgrowth happened partly because of the ecological regime shift and partly because a smaller number of people wanted to retrieve the weeds. The lack of, and a necessity for, developing community capability and holistic solutions for sustainable reuse and adaptive management of aquatic weeds were set as a revised working hypothesis. A transcend in the perception of the problem was necessary.

Based on this assessment, a realistic solution was co-created through group dialogues. In the first two workshops, participants yielded divergent ideas, such as using composted weeds for planters at school, gifted by graduates to new pupils, or creating a herbarium using endogenous weeds at school. The third and fourth workshops culminated in the idea to create a local e-point system, called *Biwa Point*, to acknowledge voluntary conservation activities, including aquatic weed conservation, based on K-san’s original idea.

**Fig. 9:**
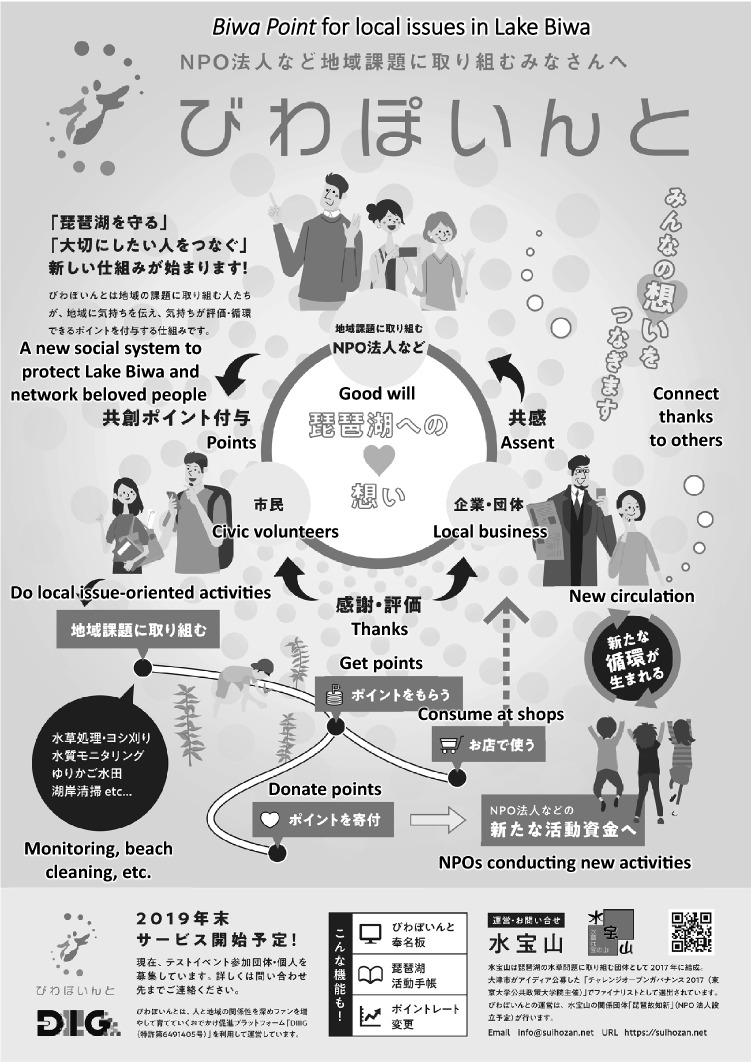
Flyer to promote the *Biwa Point* system with some English translation. Courtesy of NPO *Biwako-Chishin*

In the *Biwa Point* system (Fig. 9), companies and individuals were expected to donate *Biwa Points* to a local conservation initiative, which organizes conservation activity such as beach cleaning to remove drifted weeds. At the end of a conservation event, the organizer gives *Biwa Points* to participants, such as local inhabitants, students, or other external volunteers, as gratuity. The rewarded participants may use *Biwa Points* at local partner shops, but they are encouraged to donate *Biwa Points* to other conservation initiatives. This reinvestment is intended to enable the “circulation of goodwill.”

The *Biwa Point* system is managed by an NPO called *Biwako-Chisin* (meaning learn from old things to create new things in Lake Biwa), founded in October 2019. This NPO also hosts a portal website (https://biwako.info/) to disseminate information associated with the environment and local communities in the Lake Biwa area. *Biwako-Chisin* held a trial event for *Biwa Point* to support beach cleaning at Manohama in November 2019 (Fig. 4c) to prove that the system was effective. As of January 2021, it is preparing for the official launch of the *Biwa Point* system with local business partners.

## Discussion: community capability, social innovation, and ethical matters

### Community capability development through the circulation of goodwill

Through adaptive and abductive thinking in the multi-actor workshops, the circulation of “goodwill” was identified as a new working hypothesis to develop community capability. Circulation of *Biwa Points* will establish a non-monetary value of “goodwill” circulation with the aid of information and communication technologies. When the circulation of goodwill settles in the local communities, there will be an increased number of people who regard the aquatic weed problem as their concern. This socio-psychological transformation will reduce the number of claims concerning the washed weeds and will increase the frequency and number of participants in local environmental conservation activities such as beach cleaning. This circulation of goodwill can be explained by the theory of area capability as a proxy of community capability. Area capability is defined as follows:

“Given the emphasis of recent regional development activities on utilizing local characteristics, the scale and types of resources used and the nature of the activities are extremely diverse. That said, we found that efforts which have been able to continue sustainably and expanded in scope share the following elements: (1) A local community uses resource unique to the region; (2) Resource users understand the importance and take care of the environment that supports the resources used, and (3) A balance is struck between using and caring for resources and the supporting environment, which is evaluated by outside entities” (Ishikawa and Watanabe 2015, pp. 1-2).

According to this theoretical framework, aquatic weeds are considered a unique resource of Lake Biwa. The value of recycled weeds was rediscovered through the collaborative actions of local actors and external entities such as research experts and civic engineers. *Biwa Point* as a token of goodwill and the portal website will serve to enhance the socio-ecological capabilities of the area. A balance between using and caring for aquatic weeds to be grown and regenerated will be monitored and evaluated in the process of adaptive management not only by local governments but also by the NPO-based community.

In the circuit of community capability development, two notions were discovered regarding the scale-up of activities. The first notion was “a small-scale circulation” of composted weeds in a local community, such as the activities of Y-san and M-san in Manohama, to be transformed to “a larger-scale circulation” using *Biwa Point*. This transformation requires further work to attain an autonomous and sustainable business deployment. Financial and personnel constraints exist, including because of the current economic stagnation due to the COVID-19 pandemic.

The second notion was that the *Biwa Point* system may work to transcend the target issues from the aquatic weed problem to broader socio-ecological issues in Lake Biwa, including alien fish, plastic waste, and endangered phragmites wetland. In this regard, *Biwa Point* may attract a wider range of local inhabitants, tourists, shops, and restaurants into community-based environmental conservation programs.

### Community-based social innovation

This three-year action research project was characterized by a citizen-driven initiative for environmental governance (Zapata Campos and Zapata 2017, pp.1055–1056). Social networks that emerge from collaborative science and learning projects can foster opportunities to enhance socio-ecological resilience (Paolisso et al. 2019, p.109). Civic members recognized local socio-ecological problems to be solved by themselves and co-created a new, autonomous, and sustainable solution as a community-based innovation (Füller et al. 2006, p.60) to address a real-world problem.

Considering the characteristics of Japanese communities (Sect. 1.2), social innovations in the Japanese context are driven by a civic movement to reinforce governance through guaranteeing administrative transparency and accountability rather than a development of social relationships between actors from various backgrounds as in the Western context (Gerometta et al. 2005, p.2015; Hope Institute 2014, p.273).

In the light of this understanding of community-based innovation, our action research implies the following: first, the multifaceted review revealed that aquatic weeds were currently regarded as a nuisance, although they were historically counted as a valuable resource. This value was revived during the prefectural program. Nevertheless, the actors addressing the problem were limited to public authorities, and local inhabitants relied on them according to the questionnaire survey. In this regard, the multi-actor workshops added a new value to seek “a sustainable relationship” with aquatic weeds, which became a fundamental concept of *Biwa Point* as a platform to circulate “goodwill.”

Being different from a normal community ruled by a single principle of behavior, such as volunteerism or market economy, our interviews revealed that individual actors possessed a range of different values and principles of behavior. In this context, the workshops created new relationships among individuals and groups to form an inclusive and self-standing community. However, further development is necessary to reach the phases of “up-scaling and diffusion” and “systemic change” in the social innovation process (Murray et al. 2010, pp.12–13).

### Ethical issues between research experts and local actors: lessons learned

In our fundamental thinking, the role of external research experts in this social innovation was not as a leader of activities but as “an escort” of local actors to academically verbalize and theorize what was happening in the area. The commencement of practical research triggered the emergence of the local community. As the research continued, more conversations with *Suihōzan* members and other local actors ensued. Such interactions stimulated local civic actors to participate in community development for their own sake, and the authors were able to shift from leading the project to supporting civic actions.

During this practical research, we facilitated community capability development through financial, theoretical, analytical, and social aids. First, the research funding itself was a significant support to develop a community, and a good reason to ask local actors to participate. Second, the adaptive and abductive approach was applied at a theoretical level to realize a civic initiative to autonomously address a local socio-ecological problem. Third, the results of the multifaceted academic surveys became a reliable information source to develop a solution. Fourth, we prepared a platform for local actors to share problems and co-create solutions with a transcended view, which resulted in implementing *Biwa Point* as a system to circulate goodwill.

However, this is not a fantastic success story, but a real story of bewilderments and lessons learned. It has become evident that social practice in collaboration with diverse societal actors cannot always move ahead as originally planned. For instance, W-san repeatedly warned that a “halation” (in his words) might occur. After three years it became evident that local actors may become discouraged by researchers who publish the knowledge produced during fieldwork as “our own achievement.” The trust of local actors toward researchers may also be endangered by researchers’ publication of inconvenient truths or undesired interpretations.

This discourse of “halation” generated a social dilemma for us. When researchers became overly concerned with preventing “shadow conflict,” situations cannot be described as they truly are. The questionnaire, designed in collaboration with local governments, had to exclude some important socio-economic indicators, such as profession and household income, to protect human rights, although these proxies for income effect were essential for WTP analysis at the expected quality level of high-ranked journals in environmental economics.

Moreover, a gap in the accounting regulations between the private funding agency and the public research institution made collaboration with local private business partners difficult. Some *pro bono* engineers and workshop facilitators were disappointed and felt unfairly recognized by the low rate of reward. The fixed rate of reward offered by the public organization excluded travel hours from labor hours and was approximately one-third of the market rate for freelancers. This occurred because the incentives for researchers––academic publication and promotion––would not apply to civic volunteers with different motivations. Even voluntary work for public improvement should properly be acknowledged.

## Conclusion and future directions for citizen-driven environmental governance

This paper presented our transdisciplinary action research with an adaptive and abductive approach to facilitate developing community capability to address the excessive growth of aquatic weeds in the south basin of Lake Biwa. The initial multifaceted reviews revealed that the problem was scientifically explained by an ecological regime shift, but it was also rooted in historical and socio-psychological contexts. The postal questionnaire survey indicated that most of the unengaged public relied upon public authorities for measurement, and therefore need for capacity building for community-based conservation was identified. These findings were synthesized and assessed in multi-actor workshops to develop the *Biwa Point* system to promote and acknowledge voluntary conservation activities in Lake Biwa. In our transcended hypothesis, it may serve well to develop community capability for ecological conservation and sustainable development in the area, although connecting small-scale activities, such as what Y-san and M-san are doing in Manohama, to a large-scale platform remains as a future task.

We hope that our action research in Lake Biwa becomes a leading case of environmental autonomy to activate “shadow communities” (Sect. 1.2). Our experience was an early exploration of ethical, legal, and social issues (ELSI) in socio-ecological practical research. This experience will be delivered to social entrepreneurship communities on the occasions of Civic Tech Forum and Code for Japan Summit, as well as international communities of environmental conservation including the International Lake Environment Committee. These disseminations will laterally spread the platform and know-how to areas in need of community-based conservation, particularly for Nakaumi (Tottori and Shimane, Japan), Kasumigaura (Ibaraki, Japan), and Laguna de Bay (the Philippines), where the excessive growth of aquatic weeds appear to be a socio-ecological problem. Such dissemination will advocate citizen-driven initiatives as follows.

Regarding implication for policy-making, new citizen-driven initiatives for environmental governance are expected to transform socio-ecological policies in the future. Particularly in the rural areas of Japan, decreasing tax income due to depopulation and deindustrialization is reducing public services offered by governmental agencies. Therefore, it is necessary to transform government-dependent environmental conservation into citizen-driven and sustainable ones, to balance environmental conservation and the well-being of inhabitants in a depopulated society. Hence, public authorities need to empower civic members so that they regard socio-ecological problems as their own concerns and take action.

## Supplementary Information

Below is the link to the electronic supplementary material.

Supplementary file1 (DOCX 20 kb)

## Declarations

### Availability of data and material

The summary of the questionnaire survey is available at http://www.chikyu.ac.jp/e-rec/BiwakoSurvey2018Results.pdf (in Japanese). An English translation of the questions mentioned in the text is attached as supplementary material. The primary survey records are physically reposited in the RIHN. De-identified data will be available on request to the corresponding author.
