# Supplementary material for: Community capability building for environmental conservation in Lake Biwa (Japan) through an adaptive and abductive approach
Source: Socioecol Pract Res. 2021 Mar 29;3(2):167–83. doi: 10.1007/s42532-021-00078-3 (PMC8005670; doi:10.1007/s42532-021-00078-3)
Supplement: Supplementary file 1 — Supplementary file1 (DOCX 20 kb) [file 42532_2021_78_MOESM1_ESM.docx]

Appendix: English translations of the questions and results mentioned in Section 3.3

Source: <http://www.chikyu.ac.jp/e-rec/BiwakoSurvey2018Results.pdf>

Q3. How much did you spend in one time leisure activity in Lake Biwa last year?

(Mean: JPY 2,806)

Q4c. I am benefited from Lake Biwa.

1. Strongly agree (63.1 %)

2. Agree (28.3 %)

3. Neither agree nor disagree (6.4 %)

4. Disagree (1.9 %)

5. Strongly disagree (0.3 %)

Q4f. The alien fish populations such as large-mouth bass and blue gill in Lake Biwa should be removed by the governmental sector.

1. Strongly agree (33.7 %)

2. Agree (33.5 %)

3. Neither agree nor disagree (26.5 %)

4. Disagree (4.2 %)

5. Strongly disagree (2.1 %)

Q6a. Did you know that aquatic weeds caused bad odors when washed ashore?

0. No, I did not know it. (21.7 %)

1. Yes, I knew it. (78.3 %)

Q8a. Did you know that Shiga Prefectural Government mowed overgrown aquatic weeds?

0. No, I did not know it. (21.6 %)

1. Yes, I knew it. (78.4 %)

Q8b. Did you know that Shiga Prefectural Government composted mown aquatic weeds?

0. No, I did not know it. (53.6 %)

1. Yes, I knew it. (46.4 %)

Q8c. Did you know that Shiga Prefectural Government distributed composted weeds at free of charge?

0. No, I did not know it. (80.5 %)

1. Yes, I knew it. (19.5 %)

Q8d. Did you know that Shiga Prefectural Government supported technological development to prevent excessive growth of weeds?

0. No, I did not know it. (77.0 %)

1. Yes, I knew it. (23.0 %)

Q14. Given that a “Lake Biwa aquatic weed measurement project” launched to appropriately manage the volume of aquatic weeds in Lake Biwa, would you like to donate X yen annually?

Variable X was randomly set to 100, 300, 500, 1000, 2000, 3000, and 5000.

1. Yes (44.2 %)

2. No (55.8 %)

Q23. What is your age group?

1. 20 to 29 years old (4.0 %)

2. 30 to 39 years old (9.7 %)

3. 40 to 49 years old (14.2 %)

4. 50 to 59 years old (14.5 %)

5. 60 to 69 years old (24.0 %)

6. 70 to 79 years old (23.6 %)

7. 80 years old or elder (10.0 %)

Q24. What is your gender?

1. Male (49.8 %)

2. Female (49.5 %)

3. I don’t want to answer (0.7 %)

(End of the document)
